# Self-knowledge as a Result of the Embodied and Social Cognition

**DOI:** 10.3389/fpsyg.2019.01679

**Published:** 2019-07-17

**Authors:** Anita Pacholik-Żuromska

**Affiliations:** Nicolaus Copernicus University in Torun, Torun, Poland

**Keywords:** self-knowledge, embodiement, externalism, self-illusions, social cognition

The claim that a cognizer needs to act with the environment to gain knowledge about the world is trivial. No more does the claim sound trivial than when it is said that the cognizer^1^ also needs to interact with the environment to know himself, i.e., to gain self-knowledge (SK), defined generally as the subject's knowledge of his mental states, such as feeling, beliefs, or desires (cf. Peacocke, 1999). But why should the cognizer interact with the external world to know the content of his states if they are given to him directly by introspection? In this paper, I support the thesis that to meet the requirements put on SK as knowledge (i.e., as justified, true belief), it must be both embodied and social. Otherwise, the subject has no tool to correct his false beliefs about himself since he is simply unaware that they are false. The vision of such a self-blind subject seems not quite optimistic; hence, in this article, I would like to investigate certain solutions which could help in the argumentation against such vision.

The traditional account of SK separated the cognizer from the influence of other subjects by giving him the first-person-authority grounded on his privileged access to his internal psychological states. On such account, a society consisted of individual minds, interacting however with one another, but with no access to others' minds. On the early stage of computationalism, the already classic paradigm in cognitive science, an intuitive approach to SK was the one according to which a cognizer knew his own mental states by virtue of their appearance in mind (Haugeland, 1987; Guttenplan, 1994; Dretske, 1995). SK was then characterized by the propositional form of “I believe that I believe that *p*.” An explanation could easily be formulated with the nomenclature of computationalism by saying that to know himself, a subject needs to present two abilities (or in terms of functionalism: dispositions): to have a concept of I/Me to ascribe the attitude to oneself as to the subject of the experienced state, and to have a concept of an attitude such as BELIEF or DESIRE in order to identify the mental state in which he is (cf. Peacocke, 1992). If the concepts were understood as representations falling under computational operations (Fodor, 1998, 2000), then the SK also had a representational form composed of two basic representations: the one of I and the other of an experienced phenomenal state such as pain or belief (Newen and Vosgerau, 2007). The computability of SK, also called information processing, was determined by algorithmic processes on representations (Dretske, 1981; Fodor, 1987, 1991; Leake, 1995; David et al., 2004; Miłkowki, 2017).

On such computational account of SK, a subject was closed in the internal loop of self-representational mind, which needed no non-neural body to gain the knowledge about itself. One of the newest examples of such an internalistic model of SK is the Epistemic Agent Model (EAM,) formed on the level of conscious processing and representing its owner as an individual capable of keeping autonomous epistemic self-control, i.e., monitoring and voluntary modification of his own mental states (Metzinger, 2017, p. 8). The components of EAM are two smaller models: a model of an entity exerting control (the self) as well as a model of the satisfaction conditions of the specific mental action and the asymmetric dynamic relation connecting these two models, the one which can be interpreted simply as an intentional attitude toward a content of the mental state such as belief (cf. Metzinger, 2017). All the components are internal and based only on the neural information processing.

The subject (self) aiming at some action in the world first needs to be aware of the belief according to which he acts. To know this belief, he needs to be equipped with the model of himself as the subject having that belief. Therefore I interpret the EAM as a model of SK. Although this model does not include external elements (which are needed in the conception of the social and embodied SK) it points to a very important constituent of SK, namely the minimal phenomenal selfhood—the subjective experience of being a self. The processes responsible for physical self-specification are neuronal and hence internal. Basically, these are both homeostatic regulation as well as proprioception, which is understood as sensorimotor integration (Christoff et al., 2011, p. 104). They underlie higher level processes giving rise to self-experience. This self-experience is a fundament of EAM and, hence, SK. The constitution of SK as relying on the constitution of the self is crucial here. On the one hand, the development of self-experience constitutes a necessary element of SK, but on the other hand, it is the source of errors in self-cognition.

The errors in self-cognition are reported in many empirical studies: Rubber Hand Illusion (RHI), Full Body Illusion (FBI), or Body Swap Illusion (BSI) have shown that the perception of self-location and first-person perspective can be experimentally influenced and changed (Lenggenhager et al., 2007; Blanke and Metzinger, 2009; Ionta et al., 2011; Aspell et al., 2012), and that certain dimensions of minimal phenomenal selfhood can be manipulated (cf. Limanowski, 2014, p.1). The cases of experiencing a phantom (*de facto* missing) limb as still belonging to the body (Ramachandran and Blakeslee, 1998; Ramachandran and Altschuler, 2009; Case et al., 2010; Ramachandran et al., 2011) well exemplify lack of resistance to an error in self-cognition. The examples involving self-illusions explicitly show that we can artificially induce the experience of self-location and ownership from the outside to evoke a false self-identification, and hence, to create a false content of SK. The newest empirical findings show the connection between impairment of the self in Schizophrenia (SZ) and Autism Spectrum Disorders (ASD) accompanied by disturbances during interaction of those affected by the abovementioned mental condition with the social environment. In these cases, a subject possesses either a sharper self-others boundary which extends beyond the norm (ASD) or has weaker distinction (SZ) (Noel et al., 2017). The experiments with FBI involving ASD patients showed that the patients do not experience FBI as intensively as the healthy subjects do (Mul et al., 2019). The conclusion therefore drawn was that the multisensory integration, which constitutes the base for the minimal phenomenal selfhood formation, may be related to deficits in social functioning.

The abovementioned cases indicate the connection between the internal subjective sphere with the external sphere of the social, without giving up the role of the body in the constitution of the self. The question of SK is the question of how the body (something private and individual) interacts with the world (public and social). According to this issue called *body-social problem* (Kyselo, 2015), the social interaction relies on the tension between what is objective and what is subjective in cognition expressed in terms of distinction and participation (Kyselo, 2015). Self-cognition can be formed from the bottom up, as the basic representation of the subject as an individual distinct from other entities, but also it is shaped top-down through a subject's participation in joint actions. Both, distinction and participation lead to the development of the cognizer's beliefs as belonging to him as an individual entity with privileged access to his own states and first-person authority. They both are complementary components of the process of the cognizer's constitution as an autonomous individual in the process of continuous balancing between what is his own and what is social (cf. Kyselo, 2015).

The uniqueness of human cognition is characterized by the ability to participate with others in collaborative activities with shared goals and intentions. This ability is the so-called shared intentionality defined as an ability to share the mental states (e.g., beliefs) of others owing to the ability to represent these states. The shared intentionality can be interpreted as a reasonable conscious participation (in opposition to unreflective imitation) in social practices (Tomasello and Rakoczy, 2003; Tomasello et al., 2005). It helps to develop one's own self exemplified in the set of beliefs constituting SK. Two-year-old children are ready to understand others as intentional agents, but by age four, show the ability to read others' minds skillfully enough to be able to look from others' perspectives and understand that others can have beliefs different from their own (Baron-Cohen et al., 1985; Tomasello and Rakoczy, 2003; Tomasello et al., 2005). The ability to take the perspective of others—to think like others—to understand that others can have different beliefs is the symptom that a child has developed the theory of mind, i.e., accepts that others have their own individual minds distinctive from that of a child. This is one of the milestones in the development of SK.

Due to the conception of embodied and social SK self-cognition is the result of the body interactions with the world (Figure 1). Mind is not only “in the head” but also “in the body.” This general idea was presented by Seth (2015) and is based on empirical research on how self-experience emerges, or how the phenomenal selfhood is constructed. For the body-world interaction to be effective, the organism must present adequate abilities to control the body. These include, among others, the sense of ownership and self-identification (Seth, 2015, p. 11). The integration of bodily information in the form of bodily awareness is required because in this manner the brain creates the body model as a whole (Seth, 2015, p. 11). The self is thus an effect of interoceptive, exteroceptive, and proprioceptive sensory stimuli (Seth, 2015, p. 12). The interaction between interoceptive and exteroceptive signals is significant here (Seth, 2015, p.13), which means that, as an essential component, the self-model must also contain an external element, whose presence allows the constitution of this model. In such an externalist model of the self, an action will be a tester of SK and it will be a verifier of the beliefs concerning the subject's own states, showing that the subject in specific cases such as RHI can be wrong about the perceived object as belonging to his body, although the first information is about its integration within the body. Worth emphasizing is the fact that the presented internal and external models are based on the mechanism of predictive coding, showing that the same mechanism can underlie different models. Predictions running in the brain allow to “properly read” the current states of the world on the basis of a sensory input for the purpose of performing an appropriate action (Friston et al., 2009). I think, however, that it works properly only in the external model of SK, owing to the probability which increases after the interaction of the subject with the environment. The interaction with the world (action performing) serves as a tester of sensory input (Seth, 2015).

**Figure 1:**
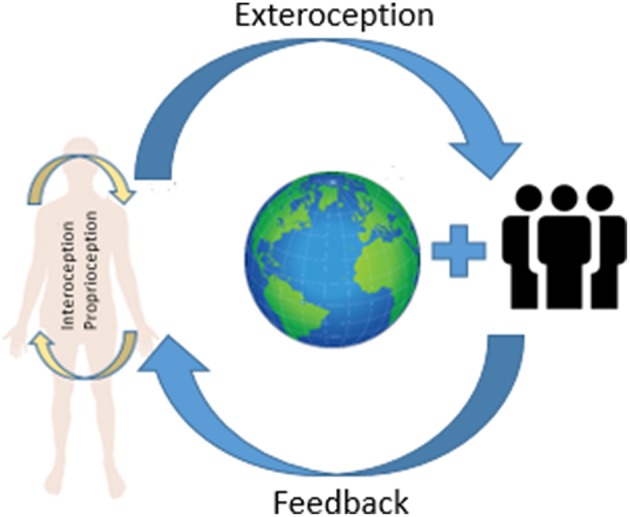
Factors influencing SK.

As it has already been said, the empirical evidence shows that the cognizer may be wrong about his experienced states. If an error arises on the basic level of information processing, for instance, an error in proprioception where the minimal phenomenal self is constituted, it is inherited by consequent levels (i.e., from sub-personal neuronal level via phenomenal up to the level of propositional mental content) until the false information appears in self-consciousness, giving the subject a wrong representation about his state. The social element constituting SK is the answer to this problem.

The social constitution of SK allows us to step out from the first-person perspective and take the third-person perspective by judging the reliability of the beliefs about the subject's own mental states. This ability opens the mind to the possibility that the cognizer can be wrong about the content of the experienced state. Although bottom up processes determine the inheritance of errors in self-experience, SK prepares us for being mistaken about our own mental states.

## Author Contributions

The author confirms being the sole contributor of this work and has approved it for publication.

### Conflict of Interest Statement

The author declares that the research was conducted in the absence of any commercial or financial relationships that could be construed as a potential conflict of interest.

## Footnotes

^2^The terms “cognizer” and “subject” are used interchangeably.
